# Finite Element Analysis of Unilateral versus Bipedicular Bone-Filling Mesh Container for the Management of Osteoporotic Compression Fractures

**DOI:** 10.1155/2022/6850089

**Published:** 2022-02-24

**Authors:** Hui Lu, Qichuan Zhang, Fan Ding, Qimei Wu, Rong Liu

**Affiliations:** ^1^School of Medicine, Wuhan University of Science and Technology, No. 1, Huangjia Lake University Town, Wuhan 430065, China; ^2^Department of Orthopedics, Puren Hospital affiliated to Wuhan University of Science and Technology, 1 Benxi Road, Wuhan 430081, China; ^3^Institute of Medical Innovation and Transformation, Puren Hospital affiliated to Wuhan University of Science and Technology, 1 Benxi Road, Wuhan 430081, China; ^4^Department of Radiology, The Second Affiliated Hospital of Army Military Medical University, Chongqing 400037, China; ^5^Wuhan Liu Sanwu Hospital of Traditional Chinese Medicine, Xinzhou District, Wuhan, 430081 Hubei Province, China

## Abstract

The effect of unilateral and bilateral bone-filling mesh containers (BFC) on osteoporotic vertebral compression fracture (OVCF) was analyzed by the finite element method. The CT scan data of the T12-L2 vertebral body were obtained from a healthy female volunteer with no history of lumbar spine injury or obvious abnormality of vertebral body morphology. The normal finite element model of the T12-L2 vertebral body and the finite element model of osteoporosis were established, and the models were validated. The L1 in the normal model of the vertebral body was used to simulate the vertebral compression fracture, after which the unilateral and bilateral BFC were simulated to establish models representing the two surgical approaches. We analyzed changes in the deformation and von Mises stress in vertebral bodies and intervertebral discs in the two models under seven working conditions (axial direction, anteflexion, rear protraction, left-side bending, right-side bending, left rotation, and right rotation) and found that the unilateral and bilateral approaches are biomechanically comparable, with no statistical difference between the two overall models.

## 1. Introduction

Osteoporotic vertebral compression fracture (OVCF) is a common orthopedic disease in the elderly, which is characterized by bone mass reduction and microstructure degeneration. OVCF is one of the most common complications of osteoporosis [1]. The increase in bone fragility can lead to osteoporotic vertebral compression fractures. Vertebral compression fractures usually occur at the thoracolumbar junction (T11-L2 level), which can lead to kyphotic deformity at the site of spinal fracture and, in severe cases, affect normal work and quality of life [2]. At present, the primary surgical treatments used are percutaneous vertebroplasty (PVP) and percutaneous kyphoplasty (PKP). PKP is a safe, effective, and minimally invasive surgery that can strengthen and stabilize the diseased vertebra and is thus widely used in treatment of the disease [3]. Studies have shown that the leakage rate of bone cement after PKP is 4.8%-39% [4]. The bone-filling mesh container (BFC) has similar surgical efficacy in the treatment of OVCF compared to PKP, but it has the additional advantages of short operation time and low bone cement leakage rate [5]. The bone-filling mesh can limit the injected bone cement to the container, significantly reducing the leakage of bone cement [6].

The mechanism of action of BFC and the mechanical characteristics of movement after bone cement implantation have been the subject of extensive research. For example, the finite element method has been used to simulate the compression fracture of the L1 vertebral body and to study the stress distribution and magnitude of L1 vertebral body under five different loads (vertical, anterior curvature, posterior extension, lateral bending, and torsion) [7]. The finite element method has also been used to construct a three-dimensional (3D) total spine model to explore the development mechanism of compression fractures [2, 8].

Clinically, BFC surgical approaches are divided into two approaches: unilateral and bilateral. There is currently no consensus on which approach is better. While evidence suggests that there is no significant difference in efficacy between the two approaches, the unilateral approach is preferable when taking into account operation time, side leakage of bone cement, and radiation exposure of personnel during the operation [9]. However, no study has yet compared the unilateral and bilateral BFC approaches from a biomechanical perspective. To address this knowledge gap, a 3D model of the human T12-L2 vertebral body was established based on CT scan images, and the Ansys finite element analysis was used to simulate the actual stress conditions of lumbar spine under seven working conditions (axial direction, anteflexion, rear protraction, left-side bending, right-side bending, left rotation, and right rotation) after unilateral and bilateral BFC surgical approaches. The biomechanical effects of the two BFC approaches in OVCF treatment were compared, providing a theoretical basis for practical clinical application.

## 2. Materials and Methods

### 2.1. Patient Conditions

The scanning object was a 65-year-old female volunteer with no significant abnormalities in vertebral body morphology. The T12-L2 CT data were obtained. The volunteer was not complicated with cardiovascular and cerebrovascular diseases, severe liver or renal impairment, or mental illness, and informed consent was signed by the patients and their families. The experimental protocol was approved by the hospital ethics committee. The whole lumbar vertebral body was scanned with Siemens 64-slice spiral CT at 140 kV, 200 mA, and 0.625 mm thickness. The CT data were extracted in a 512 × 512-pixel DICOM format.

### 2.2. Establishment of 3D Model of Normal and Osteoporotic T12-L2

**Figure 1:**
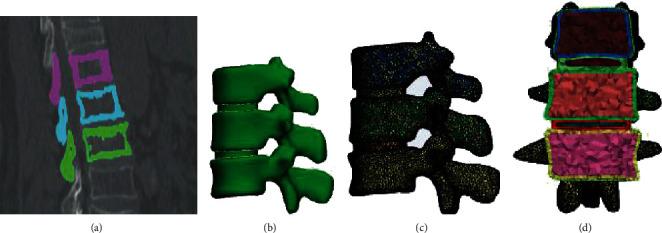
Establishment of a 3D model of the normal lumbar spine (T12-L2). (a) Threshold extraction. (b) Generate 3D models. (c) Generate surface meshes. (d) Generate a volume grid.

The volunteer CT data were imported into Mimics 20.0 (Materialise NV, Leuven, Belgium, 2017) for 3D reconstruction. The threshold division was carried out according to the gray value of each tissue structure, and different parts of the lumbar spine models were established. Finally, the geometric models of T12-L2 vertebral body and intervertebral disc were established and derived in a stereolithography (STL) format [10]. The 3D model of the lumbar spine was then imported into 3-matic 12.0 (Materialise NV), and the surface of the lumbar spine was smoothed through mesh diagnosis, processing of bad mesh, surface parameter fitting and other operations, and generating the finished model of body mesh in the lumbar spine (Figure 1).

### 2.3. Setting Material Properties and Validation of the Model

In order to simplify calculation processes, only the mechanical properties of the bone structure within the elastic range were considered, and the isotropic, uniform, and continuous elastic material model was used to characterize the bone structure. The specific material parameters are shown in Table 1 and are based on previous reports [11–14]. After completing the assignment of material attributes, the lower endplate of the L2 vertebral body was set as the fixed surface, and loads were allocated from the upper endplate of the T12 vertebral body according to the theory of three columns of the spine, in which 85% of the weight load is carried by the anterior midcolumn, and about 15% is carried by the elements behind the vertebrae [15–18]. A vertical downward load of 500 N was applied to all models, along with a torque of 7.5 Nm, to simulate lumbar axial direction, anteflexion, rear protraction, left-side bending, right-side bending, left rotation, and right rotation [19]. The range of activity in our model was consistent with standard values in the literature [17, 18, 20].

### 2.4. Establishment of the Operative Finite Element Model

**Figure 2:**
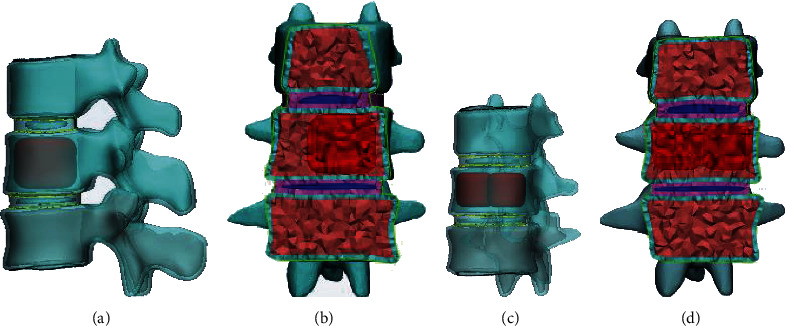
Surgical models of group M1 and group M2 after bone cement injection. (a) M1-side view; (b) M1-front view; (c) M2-side view; and (d) M2-front view.

After bone cement reinforcement, the anatomical geometry of a vertebral body with compression fracture is similar to that of a normal vertebral body [14]. However, to simplify the model, the height of the fractured and normal vertebral bodies was set to the same values. Compression fractures of the L1 vertebra in the normal finite element model were simulated. The model uses a globular type of structure simulation for the bone cement. The percentage of bone cement volume was about 23% (8.4 mm^3^ bone cement volume, 36.7 mm^3^ L1 vertebral body volume). Distribution of the reinforcement material was located inside the vertebral body, not in contact with the surrounding cortical bone or upper and lower endplates, as this shape was similar to the distribution of the implant seen on the X-ray of the treated patient [21]. A unilateral BFC model (M1) and a bilateral BFC model (M2) were established (Figure 2). After completing the assignment of material attributes, the lower endplate of the L2 vertebral body was set as the fixed surface, and loads were allocated from the upper endplate of the T12 vertebral body according to the theory of three columns of the spine, in which 85% of the weight load is carried by the anterior midcolumn, and about 15% is carried by the elements behind the vertebrae [15–18]. A vertical downward load of 500 N was applied to all models, along with a torque of 7.5 Nm, to simulate lumbar axial direction, anteflexion, rear protraction, left-side bending, right-side bending, left rotation, and right rotation. After loading boundary conditions, finite element analysis was carried out on the model.

### 2.5. Observational Index and Statistical Analysis

Changes in the deformation and von Mises stress were analyzed for T12, L1, and L2 centra, and for T12-L1 and L1-L2 intervertebral discs, under seven working conditions. Results were compared between the M1 (unilateral) and M2 (bilateral) BFC models. The Shapiro-Wilk goodness-of-fit test was used to test the sample distribution. A paired *t*-test was used to analyze the normal quantitative data of the two groups, and the Mann–Whitney *U* test was used to analyze the skewed data of the two groups. The descriptive content gives mean, standard deviation, and median (quartile). All analyses were performed using SPSS 20.0; *p* < 0.05 was considered statistically significant.

## 3. Results

### 3.1. Validation of Lumbar Finite Element Models

**Figure 3:**
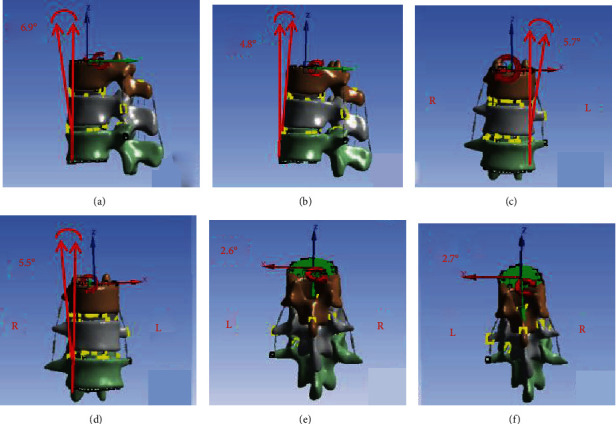
Results of validation of the model. (a) Anteflexion 6.9°; (b) rear protraction 4.8°; (c) left-side bending 5.7°; (d) right-side bending 5.5°; (e) left rotation 2.6°; and (f) right rotation 2.7°.

In this study, loading analysis was performed on normal and osteoporotic lumbar finite element models under seven operating conditions (axial direction, anteflexion, rear protraction, left-side bending, right-side bending, left rotation, and right rotation). Range of motion (ROM) was also measured under each operating condition. The results were very similar to those of previous studies [20, 22–25] (Table 2).  Figure 3 shows the validation of the finite element model.

### 3.2. Analysis of OP, M1, and M2 Models under Seven Operating Conditions

**Figure 4:**
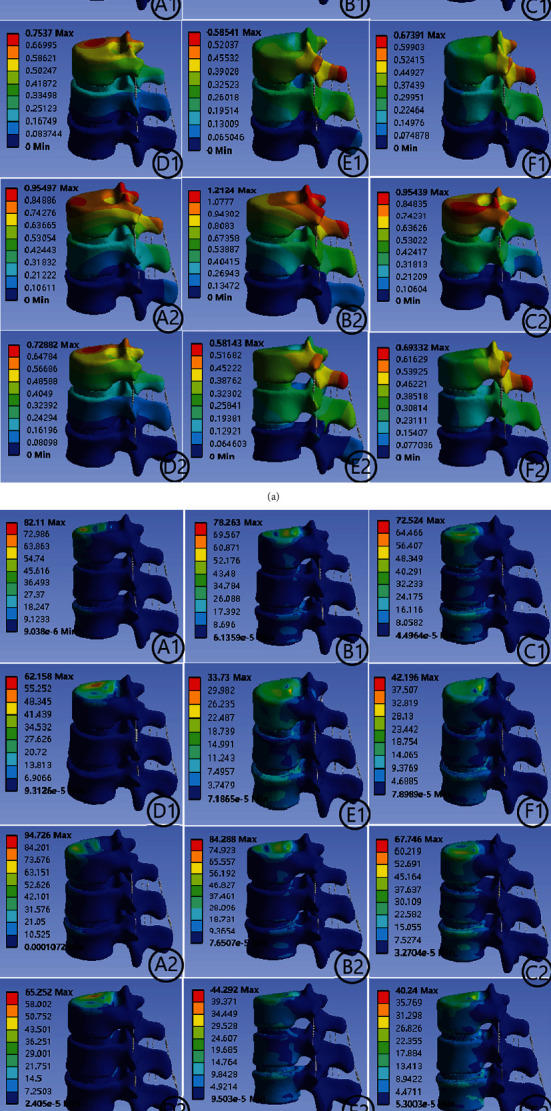
(a) The deformation of group M1 and group M2 under six conditions. (A1) Anteflexion M1 deformation. (B1) Rear protraction M1 deformation. (C1) Left-side bending M1 deformation. (D1) Right-side bending M1 deformation. (E1) Left rotation M1 deformation. (F1) Right rotation M1 deformation. (A2) Anteflexion M2 deformation. (B2) Rear protraction M2 deformation. (C2) Left-side bending M2 deformation. (D2) Right-side bending M2 deformation. (E2) Left rotation M2 deformation. (F2) Right rotation M2 deformation. (b) The von Mises stress distribution of group M1 and group M2 under six conditions. (A1) Anteflexion M1 von Mises stress. (B1) Rear protraction M1 von Mises stress. (C1) Left-side bending M1 von Mises stress. (D1) Right-side bending M1 von Mises stress. (E1) Left rotation M1 von Mises stress. (F1) Right rotation M1 von Mises stress. (A2) Anteflexion M2 von Mises stress. (B2) Rear protraction M2 von Mises stress. (C2) Left-side bending M2 von Mises stress. (D2) Right-side bending M2 von Mises stress. (E2) Left rotation M2 von Mises stress. (F2) Right rotation M2 von Mises stress.

Figure 4(a) indicates the deformation and von Mises stress distribution of group M1 and group M2 under axial direction, anteflexion, and rear protraction.  Figure 4(b) indicates the deformation and von Mises stress distribution of group M1 and group M2 under left-side bending, right-side bending, left rotation, and right rotation.

**Figure 5:**
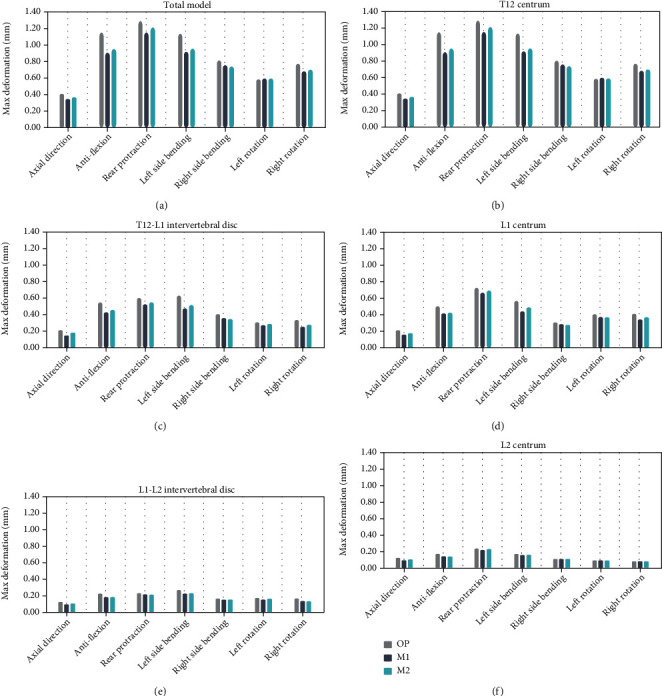
Deformation changes of T12 centrum, T12-L1 intervertebral disc, L1 centrum, L1-L2 intervertebral disc, and L2 centrum under seven operating conditions. (a) Total model; (b) T12 centrum; (c) T12-L1 intervertebral disc; (d) L1 centrum; (e) L1-L2 intervertebral disc; and (f) L2 centrum.

Figure 5 indicates the deformation changes of T12 centrum, T12-L1 intervertebral disc, L1 centrum, L1-L2 intervertebral disc, and L2 centrum under all seven operating conditions. The deformation of adjacent vertebral bodies and intervertebral discs in the M1 group and the M2 group were not significantly different. Compared to the OP (osteoporosis) group, the deformation was correspondingly smaller, indicating that injection of bone cement made the deformation of adjacent vertebral bodies and intervertebral discs smaller.

As can be seen from Table 3, T12-L1 disc deformation was significantly higher in the M2 group than in the M1 group (0.39 ± 0.14 mm vs. 0.37 ± 0.13 mm; *t* = −3.240, *p* = 0.018). However, there was no significant difference between the two models in deformation of the T12 centrum (*t* = −2.027, *p* = 0.089), L1 centrum (*t* = −2.200, *p* = 0.07), L2 centrum (*Z* = −1.000, *p* = 0.317), or the L1-L2 disc (*Z* = −1.732, *p* = 0.25). Likewise, there was no significant difference in overall deformation between the M1 and M2 groups in the finite element analysis of the thoracolumbar fracture treatment.

**Figure 6:**
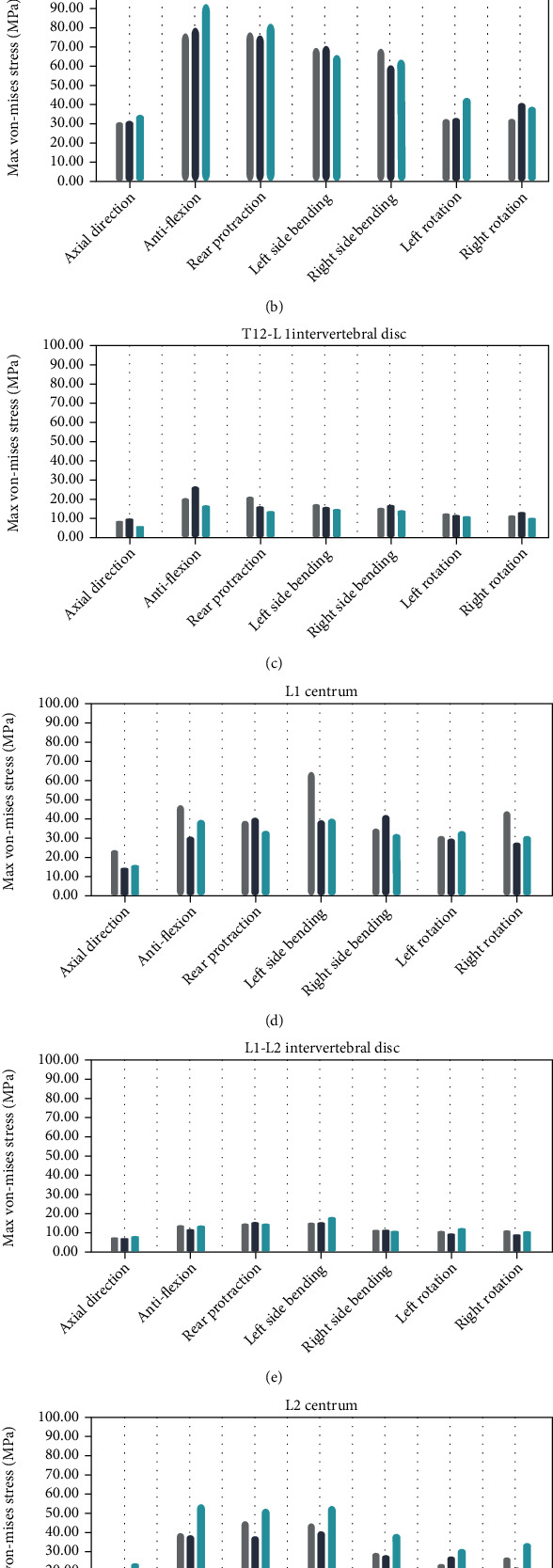
The von Mises stress changes of T12 centrum, T12-L1 intervertebral disc, L1 centrum, L1-L2 intervertebral disc, and L2 centrum under seven operating conditions. (a) Total model; (b) T12 centrum; (c) T12-L1 intervertebral disc; (d) L1 centrum; (e) L1-L2 intervertebral disc; and (f) L2 centrum.

Figure 6 indicates the von Mises stress changes in T12 centrum, T12-L1 intervertebral disc, L1 centrum, L1-L2 intervertebral disc, and L2 centrum under different operating conditions. The stress changes in adjacent discs in the M1 and M2 groups were similar to those in the OP group under all seven conditions. However, the variation of the von Mises stress in the 12 thoracic vertebrae was significantly higher in the M2 group than in M1 or OP, with the variation in M1 being slightly lower than that in OP. Changes of the von Mises stress in the L2 thoracic vertebrae groups were significantly higher in the OP group than in the M1 and M2 groups, while in the L2 vertebral groups, it was significantly higher in the M2 group than in the other two groups, with variation slightly higher in the M1 group than that in the OP group. Overall, these results indicated that the von Mises stress was mainly concentrated in the fractured vertebral body before injection of bone cement; after the injection of bone cement, the von Mises stress was mainly distributed in the adjacent vertebrae and was greater in the adjacent vertebrae after the bilateral rather than the unilateral injection of bone cement.

As can be seen from Table 4, statistical analysis showed that there was no significant difference in the von Mises stress results between the two groups of models (*t* = −1.751, *p* = 0.13). There was no significant difference in the results of the von Mises stress of the T12 vertebral body between the two groups (*t* = −1.751, *p* = 0.13). The results of the von Mises stress of T12-L1 intervertebral discs in group M1 (15.21 [11.31, 16.47] MPa) were significantly higher than those in group M2 (13.05 [9.36, 14.20] MPa, *Z* = −2.366, *p* = 0.016). There was no significant difference in the results of the von Mises stress of L1 vertebral body between the two groups (*t* = −0.082, *p* = 0.937). There was no significant difference in the results of the von Mises stress of L1-L2 discs between the two groups (*t* = −1.752, *p* ≥ 0.13). The von Mises stress of the L2 vertebral body in the M2 group (43.42 ± 12.52 MPa) was significantly higher than that in the M1 group (31.91 ± 9.74 MPa) (*t* = −6.221, *p* = 0.001).

In summary, the results showed no significant difference in the von Mises stress of the T12 vertebral body, the L1 vertebral body, or the L1-L2 intervertebral disc between the two groups. However, the von Mises stress of the T12-L1 disc and L2 vertebral body between the two groups was statistically significant. Taken together, there was no significant difference in the overall finite element analysis of the treatment of thoracolumbar fractures between the M1 group and the M2 group.

## 4. Discussion

Osteoporotic vertebral compression fracture (OVCF) is a serious health problem associated with population aging [26]. Conservative treatment often leads to many complications; therefore, surgical treatment has become the preferred method [27]. Percutaneous vertebroplasty (PVP) and percutaneous kyphoplasty (PKP) are both routine and minimally invasive surgeries which can help patients effectively relieve pain, but they cannot completely recover vertebral height [28, 29]. PKP can better restore vertebral height by using balloon dilation [30] and is favored by clinicians over PVP. However, both PVP and PKP surgery methods have the problem of bone cement leakage. In order to reduce this issue, the bone-filling mesh container (BFC) combined with PKP can be used to treat OVCF, which can not only relieve the clinical symptoms of patients and restore the vertebral height to a certain extent but also effectively reduces bone cement leakage. Currently, clinical studies have shown that bilateral and unilateral BFC have similar clinical efficacy in the treatment of OVCF, but unilateral BFC is a shorter surgical procedure than bilateral BFC, reducing both intraoperative radiation times and bone cement leakage. However, it lacks theoretical support in biomechanics.

Some biomechanical cadaver studies have been conducted to address this. One such study analyzed the efficacy of bilateral versus unilateral vertebroplasty [31]. Although the intensity of the bilateral group was significantly higher than that of the unilateral group, both groups were significantly stronger than intact vertebrae. There was no significant difference in stiffness between the two groups, and both showed stiffness similar to the prefracture state. Another such study analyzed the efficacy of bilateral versus unilateral kyphoplasty [9]. They found that unilateral and bilateral pedicle kyphoplasty were comparable in strength, stiffness, and height of the vertebral body in the repair of experimental vertebral compression fractures. The risk of lateral wedging was not greater in the unilateral group than in the bilateral group. However, the unilateral approach offers advantages over the bilateral approach in terms of the risk of pedicle intubation, surgical time, radiation exposure, and cost.

Despite the fact that the research from the perspective of biomechanics analysis of unilateral and bilateral approaches that contrast the curative effect of treatment of PVP and PKP holds, to get the data from the studied specimen, with the specimen now harder, it increases the difficulty for clinical biomechanics research. We therefore implemented biomechanical analysis in a virtual model using finite element methods. This reduced the difficulties involved in clinical biomechanical research and increased the reproducibility of the experiment.

Although the usefulness of spinal finite element analysis has been confirmed in many studies [32–36], no study has used finite element analysis to compare the efficacy of unilateral and bilateral BFC approaches in the treatment of OVCF. In this study, four T12-L2 3D finite element models (OP, M1, and M2) were established using 3D reconstruction software, finite element analysis software, and biomechanical material properties. Together, these reflected the pathological characteristics of vertebral osteoporosis and the clinical characteristics of OVCF treated by unilateral and bilateral approaches of bone cement injection into the vertebral body. These virtual finite element models provide theoretical support for preoperative planning, postoperative biomechanical evaluation, and minimally invasive technique improvement of BFC.

We found that bone cement injection resulted in smaller deformation of adjacent vertebral bodies and intervertebral discs. The stress changes in adjacent discs in the M1 and M2 groups were similar to those in the OP group under seven working conditions. In the thoracic L2 vertebral body stress change, the M2 group was significantly higher than the other two groups, and the M1 group was slightly less than the OP group stress change. The stress changes of the L2 thoracic vertebrae in the OP group were significantly higher than those in the M1 and M2 groups. In L2 vertebral body stress change, the M2 group was significantly higher than the other two groups, and the M1 group was slightly higher than the OP group. There was no significant difference in the deformation and stress distribution between group M1 and group M2 in the finite element analysis of the treatment of thoracolumbar fracture (Tables 3 and 4).

These results indicate that with the injection of bone cement, the stress of the fractured vertebra is gradually dispersed to the adjacent vertebra. Bilateral bone cement injection showed better results than the unilateral group, but both groups were significantly better than the OP group. Indirectly, the strength and stiffness of the fractured vertebrae were enhanced by the injection of bone cement, which is consistent with prior results. It also suggests an increased risk of fractures in adjacent vertebrae following surgery. This is consistent with another study showing that secondary compression fractures most often occur in adjacent vertebral bodies [37]. However, there is a lack of risk comparison between the finite element analysis of unilateral and bilateral approach BFC for OVCF.

There are some limitations of this study that should be noted. First, the current model does not effectively simulate ligaments and articular cartilage. We only simulate the intertransverse ligament, interspinous ligament, and supraspinous ligament but do not simulate the anterior longitudinal ligament, posterior longitudinal ligament, ligamentum flavum, and articular cartilage. Second, we only simulated the physiological state under seven working conditions, which cannot fully capture the range of human thoracolumbar motion. Third, our simulation of the surgical process is relatively simple. We simulate the surgical process by simply reconstructing the surgical model, while in the actual surgical process, skin thickness and the position, depth, and angle of the needle must be considered. Nevertheless, we believe that the results of this study, as well as the methods described, are a valuable reference for clinical work.

## 5. Conclusion

From the perspective of biomechanics, there is little difference between unilateral and bilateral BFC in OVCF treatment. Both can reduce stress of the fractured vertebra to a certain extent, although this leads to a corresponding increase in stress on the adjacent vertebra, thus increasing their risk of fracture. Based on our results, we believe that the unilateral and bilateral BFC approaches are equally effective in treating OVCF with respect to biomechanics. Future research should focus on incorporation of data related to articular cartilage and ligaments and include additional working conditions to more completely capture the realistic complexity of movement after BFC operation, providing further theoretical support for clinical work.

## Figures and Tables

**Table 1:** Material properties of the osteoporotic T12-L2 finite element model.

Material	Elastic modulus, *E* (MPa)	Poisson ratio, *μ*	Stiffness coefficient	Status
Cortical bone	8040 (67% normal)	0.3	—	Osteoporotic
Cancellous bone	34 (34% normal)	0.2	—	Osteoporotic
Bony endplate	670 (67% normal)	0.4	—	Osteoporotic
Posterior structure	2345 (67% normal)	0.25	—	Osteoporotic
Annulus fibers	455	0.3	—	Normal
Nucleus pulposus	0.4	0.499	—	Normal
Facet cartilage	10	0.4	—	Normal
Anterior longitudinal	20	0.3	33.0	Normal
Posterior longitudinal	70	0.3	20.4	Normal
Interspinous	28	0.3	11.5	Normal
Supraspinous	28	0.8	23.7	Normal
Ligamentum flavum	50	0.3	27.2	Normal
Intertransverse	50	0.3	15.0	Normal
Bone cement (PMMA)	3000	0.4	—	Grafting

**Table 2:** Range of motion of the finite element model of T12-L2 and comparison with the previous research result (°).

Operating condition	The present research	[20]	[22]	[23]	[24]	[25]	The present OP model
Anteflexion	6.8 ± 2.15	9.15	7.21	6.51	7.0	7.9	6.9
Rear protraction	5.0 ± 1.34	5.42	4.68	5.43	4.5	6.8	4.8
Left-side bending	5.5 ± 1.75	13.32	7.21	5.47	5.6	7.3	5.7
Right-side bending	5.3 ± 1.44	13.31	7.43	5.43	6.7	8.0	5.5
Left rotation	2.2 ± 1.42	4.21	—	2.54	3.1	2.8	2.6
Right rotation	2.5 ± 1.36	3.96	—	2.46	3.8	3.3	2.7
Axial direction	—	—	—	—	—	—	—

**Table 3:** Deformation statistical analysis results of the two groups of models in the total model, T12 centrum, T12-L1 intervertebral disc, L1 centrum, L1-L2 intervertebral disc, and L2 centrum.

	Group	Mean ± standard deviation, median (quartile)	*N*	*T*/*Z*	*p*
Total model	M1	0.76 ± 0.26	7.00	-2.027	0.089
	M2	0.78 ± 0.28	7.00		
T12 centrum	M1	0.76 ± 0.26	7.00	-2.027	0.089
	M2	0.78 ± 0.28	7.00		
T12-L1 disc	M1	0.37 ± 0.13	7.00	-3.240	0.018
	M2	0.39 ± 0.14	7.00		
L1 centrum	M1	0.39 ± 0.15	7.00	-2.200	0.070
	M2	0.41 ± 0.16	7.00		
L1-L2 disc	M1	0.15 (0.13, 0.21)	7.00	-1.732	0.250
	M2	0.16 (0.13, 0.21)	7.00		
L2 centrum	M1	0.11 (0.08, 0.16)	7.00	-1.000	0.317
	M2	0.11 (0.08, 0.16)	7.00		

**Table 4:** The von Mises stress statistical analysis results of the two groups of models in the total model, T12 centrum, T12-L1 intervertebral disc, L1 centrum, L1-L2 intervertebral disc, and L2 centrum.

	Group	Mean ± standard deviation, median (quartile)	*N*	*T*/*Z*	*p*
Total model	M1	57.57 ± 21.35	7.00	-1.751	0.130
	M2	61.71 ± 22.75	7.00		
T12 centrum	M1	57.57 ± 21.35	7.00	-1.751	0.130
	M2	61.71 ± 22.75	7.00		
T12-L1 disc	M1	15.21 (11.31, 16.47)	7.00	-2.366	0.016
	M2	13.05 (9.36, 14.20)	7.00		
L1 centrum	M1	33.25 ± 10.16	7.00	-0.082	0.937
	M2	33.46 ± 8.65	7.00		
L1-L2 disc	M1	12.05 ± 3.47	7.00	-1.752	0.130
	M2	13.17 ± 3.34	7.00		
L2 centrum	M1	31.91 ± 9.74	7.00	-6.221	0.001
	M2	43.42 ± 12.52	7.00		

## Data Availability

All data were collected from the Puren Hospital, Wuhan City, Hubei Province, China.
